# A new genus of oak gallwasp, *Coffeikokkos* Pujade-Villar & Melika, gen. n., with a description of a new species from Costa Rica (Hymenoptera, Cynipidae)

**DOI:** 10.3897/zookeys.168.2030

**Published:** 2012-01-31

**Authors:** Juli Pujade-Villar, Paul Hanson, George Melika

**Affiliations:** 1 University of Barcelona. Faculty of Biology. Department of Animal Biology. Avda. Diagonal 645, 08028 - Barcelona, Spain; 2 Escuela de Biología, Universidad de Costa Rica, San Pedro, Costa Rica; 3 Pest Diagnostic Laboratory, Plant Protection & Soil Conservation Directorate of County Vas, Ambrozy setany 2, Tanakajd 9762, Hungary

**Keywords:** Cynipini, *Coffeikokkos*, Neotropical Region, *Quercus bumelioides*, taxonomy, morphology, distribution, biology

## Abstract

A new genus of oak gallwasp, *Coffeikokkos *Pujade-Villar & Melika, **gen. n.**, is described from Costa Rica. Diagnostic characters and generic limits of the new genus are discussed in detail. The new genus includes *Coffeikokkos copeyensis *Pujade-Villar & Melika, **sp. n.**, which induces galls on stems of *Quercus bumelioides*, an endemic oak to Costa Rica, Honduras and Panama. The new species and galls are described and illustrated.

## Introduction

Nineteen species of oaks are listed for Costa Rica (Govaerts and Frodin 1998), which appear to support quite a high species richness of oak gallwasps (Hymenoptera, Cynipidae, Cynipini). There are probably more than 30 species present in the country (Pujade-Villar and Hanson 2006), but only three species, *Odontocynips hansoni* Pujade-Villar, *Andricus costaricensis* Pujade-Villar & Melika, and *Disholcaspis costaricensis* Melika & Pujade-Villar have been described thus far (Pujade-Villar 2009; Melika et al. 2009, 2011). The new Cynipini genus and the new species described here, represent the fourth known species and the fourth known genus from Costa Rica.

*Coffeikokkos* gen. n., closely resembles the genus *Cynips* L.; however, the number of antennal flagellomeres, the shape of the ventral spine of the hypopygium (among other characters), the shape and structure of the gall, differ between these two genera.

## Material and methods

Adult gallwasps were reared from galls collected on *Quercus bumelioides* Liebm. (= *Quercus copeyensis* C.H.Mull.) by Paul Hanson (details below).

We follow the current terminology for morphological structures in Liljeblad and Ronquist (1998) and Melika (2006). Abbreviations for fore wing venation follow Ronquist and Nordlander (1989), and cuticular surface terminology follows that of Harris (1979). Measurements and abbreviations used here include: F1-F15, 1st and subsequent flagellomeres; POL (post-ocellar distance) is the distance between the inner margins of the posterior ocelli; OOL (ocellar-ocular distance) is the distance from the outer edge of a posterior ocellus to the inner margin of the compound eye; LOL, the distance between lateral and frontal ocelli. The width of the forewing radial cell is measured from the margin of the wing to the Rs vein.

The SEM photographs were taken with a Stereoscan Leica-360 by Palmira Ros-Farré (Universitat de Barcelona) at a low voltage (700V) and without coating, in order to preserve the specimens. Pictures of the adult wasp habitus and wings were taken by a digital camera Canon PowerShot SX210 15 by Juli Pujade-Villar. Gall images were taken by Kenji Nishida. The images will be available from the “morphbank.com” databank.

The type material is deposited in the following institutions:

**UB** Universitat de Barcelona, Spain (J. Pujade-Villar);

**PDL** Pest Diagnostic Laboratory (the former Systematic Parasitoid Laboratory, SPL), Tanakajd, Hungary (G. Melika);

**MZUCR** Museo de Zoología, Universidad de Costa Rica (P. Hanson).

Hymenopteran parasitoids reared from the galls are deposited in MZUCR.

## Results

### Coffeikokkos

Pujade-Villar & Melika gen. n.

urn:lsid:zoobank.org:act:6DDF6EC0-5FC4-4C65-ADF1-DA8D47AAB4E0

http://species-id.net/wiki/Coffeikokkos

#### Type species.

*Coffeikokkos copeyensis*Pujade-Villar & Melika, sp. n., by present designation.

#### Diagnosis.

*Coffeikokkos* is the only known genus of Cynipini with 14–15 antennal flagellomeres in females (in some paratypes the suture between F14 and F15 is weakly indicated, but in one female it is absent and the antennae therefore has 14 distinctly visible flagellomeres). Adults of *Coffeikokkos* most closely resemble the parthenogenetic forms of *Cynips* Linnaeus species in the morphology and surface sculpture of the head, mesosoma and the shape of the ventral spine of the hypopygium. Among all known *Cynips* species, the western Palaearctic *Cynips korsakovi* Belizin most closely resembles *Coffeikokkos copeyensis*: this is the only *Cynips* species which has the mesoscutum without dense setae (present only along notauli) and the sides of the ventral spine of the hypopygium parallel, not broadened at the apex. In *Coffeikokkoss* the antennae have 14–15 flagellomeres; the clypeus is small, rounded, not emarginate ventrally; the tarsal claws are simple, with a rounded basal lobe; the lateral propodeal carinae are incomplete, not reaching the nucha, subparallel in the anterior half and strongly curved outwards in the posterior half of the propodeum; the ventral impressed area is higher than the height of the metascutellum; and the 2^nd^ metasomal tergite has very few white setae anterolaterally. In the asexual females of *Cynips* species (and particularly in *Cynips korsakovi*), the antennae have 12 flagellomeres; the tarsal claws have a narrow, acute basal lobe; the clypeus is widely emarginate ventrally and overhanging the mandibles; the lateral propodeal carinae are complete, reaching the nucha, nearly subparallel, slightly curved outwards in the middle; the ventral impressed area is 2.0 times shorter than the height of the metascutellum; the 2^nd^ metasomal tergite has numerous white setae anterolaterally. Galls of all known *Cynips* species are located mostly on the underside of leaves, while in *Coffeikokkos*they are located on stems.

#### Description.

 Asexual female with robust and glabrous body. Head broadened behind eye in anterior view, malar sulcus absent. Antenna with 14–15 flagello-meres. Mesoscutum smooth and shiny, notauli deep, complete, reaching pronotum. Mesoscutellum dull rugose with transverse depression anteriorly, scutellar foveae present but always indistinctly delimited posteriorly (in some paratypes foveae separated by weak median carina). Propodeum with incomplete lateral propodeal carinae, subparallel in anterior half and strongly divergent posteriorly. Tarsal claws simple with broad and rounded basal part. Metasoma without punctures, shiny; 2^nd^ metasomal tergite with sparse white setae laterally. Projecting part of ventral spine of hypopygium broad, longer than wide, rounded apically, with long dense subapical setae forming tuft directed backwards and reaching beyond apex of spine.

#### Etymology.

 The name reflects the shape and the colour of the growing galls which are similar to the shape of a coffee berry and the Greek *kokkos* (*κόκκος*) means “berry”.

#### Gender.

 Masculine.

### Coffeikokkos copeyensis

Pujade-Villar & Melika sp. n.

urn:lsid:zoobank.org:act:7891EF83-20C1-4238-BB2E-674B46ABA4A5

http://species-id.net/wiki/Coffeikokkos_copeyensis

#### Type material.

 HOLOTYPE female (deposited in the collection J.P-V, UB): “COSTA RICA, San José, Cerro de la Muerte, Est. Biol. Cuericí, 2600 m, ix-1997, P. Hanson" (white label), *Quercus copeyensis*, red detachable stem gall" (white label), “HOLOTYPE *Coffeikokkos copeyensis* agam ♀ n. gen & n. sp. design. J.P-V 2011" (red label). PARATYPES (12♀): 6♀ with the same data as the holotype (3♀ UB, 2♀ PDL, 1♀ MZUCR); 2♀: “COSTA RICA San José Prov., Parque Nacional Chirripó, Llano Bonito, 2600 m, 09º27'16''N 83º32'41''W, (10.VII.2010) late ix-x-2010, *Quercus copeyensis*, Red berry gall" (1♀ UB, 1♀ MZUCR); 3♀: COSTA RICA, Cartago, 4 Km NE Canón Genesis II, 2300m, i.1995, P. Hanson", “*Quercus copeyensis*, red detachable stem gall" (2♀ PDL, 1♀ MZUCR); 1♀: “COSTA RICA San José, Cerro Muerte, 6 Km N San Gerardo, 2800m, xii.1992, Hanson & Godoy (MZUCR).

#### Diagnosis.

Head with piliferous punctures; antennae 16–17 segmented with F1 longer than F2; the body smooth and shiny; notauli complete, deep; propodeal carinae incomplete, not reaching the nucha; the prominent part of the ventral spine of the hypopygium longer than broad, with parallel sides for the entire length, rounded distally, with a tuft of long subapical setae.

#### Description.

(Figs 1–13). Asexual form.

#### Length.

Female 4.1–5.1 mm (n = 12).

#### Coloration.

(Fig. 13). Body predominantly uniformly brown; following areas dark brown to black: postocciput, postgena and postgenal bridge; propleura and anterior rim of pronotum; mesonotum ventrally, mesoscutellum, subaxillular bar, metascutellum with ventral impressed area, metanotum, metanotal trough, propodeum and nucha. Scape and pedicel always brown, flagellomeres from brown to black. Coxae and femora yellowish white, tibiae and tarsi dark brown to black. 2^nd^ metasomal tergite always brown, subsequent tergites brown, in some paratypes lighter. Forewing with smoky areas; veins dark brown.

#### Head.

**Figures 1–5.**
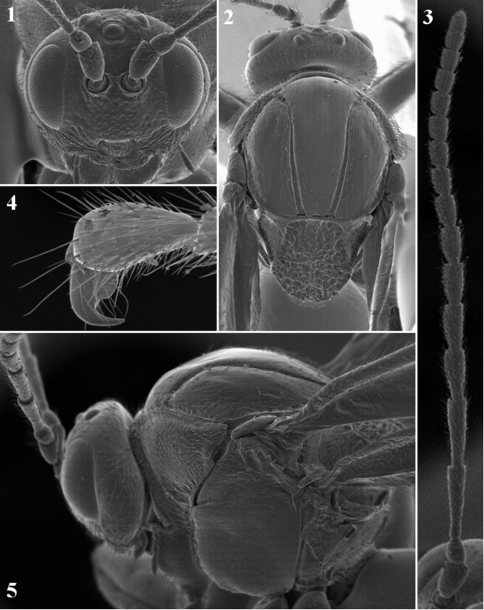
*Coffeikokkos copeyensis*, asexual female **1** head (anterior view) **2** head and mesosoma (dorsal view) **3** antenna **4** hind tarsal claw **5** head and mesosoma (lateral view).

 (Figs 1–2, 5). Narrower than mesosoma, with white sparse setae. Head ovate, 1.3–1.4 times as broad as high in anterior view and 3.7–3.8 times as broad as long in dorsal view; gena slightly broadened behind eye, smooth and shiny, punctured basally; malar space without sulcus, 0.25 times as long as eye height, with striae radiating from clypeus and nearly reaching eye margin. Lower face shiny, with deep punctures, without elevated area medially. Clypeus impressed, setose, alutaceous, rounded and slightly emarginate ventrally, medially not incised, anterior tentorial pits small, indistinct; epistomal sulcus and clypeo-pleurostomal line distinct, broad, impressed. POL:OOL:LOL=8:4:3, diameter of lateral ocellus equal OOL; frons smooth and shiny. Vertex and occiput coriaceous, with piliferous points.

#### Antenna.

(Fig. 3). 15 flagellomeres (rarely 14); F1 longer than F2, broader distally; subsequent flagellomeres progressively shorter, F15 longer than F14. Antennal formula: 6: 3.5(×2.5): 13(×2.5): 10(×3): 8: 7: 6.5: 5.5: 5: 4.5: 4: 4: 3: 3: 3: 3: 4.5. Placodeal sensilla on F3–F15.

#### Mesosoma.

 (Figs 2, 5, 9). Longer than high, dorsally concave in lateral view. Pronotum setose, with coriaceous sides and few weak carinae posterolaterally, anterolateral rim of pronotum strongly carinate, with deep invagination along side. Mesoscutum slightly broader than long in dorsal view, smooth, shiny, with sparse setae laterally and along notauli; notauli complete and deep, median mesoscutal line indistinct, absent or very short and superficial; parapsidal lines indistinct, anterior parallel lines differentiated by delicate sculpture. Mesopleuron smooth and shiny, with delicate setae. Mesoscutellum longer than broad in dorsal view, setose, uniformly dull rugose; scutellar foveae superficial, rugose, indistinctly delimited, almost confluent, with delicately rugose and shiny bottom; median (central) carina absent or present but inconspicuous. Propodeum alutaceous and setose; lateral propodeal carinae incomplete, not reaching nucha, nearly parallel and straight in anterior half and strongly divergent in posterior half, central propodeal area glabrous, smooth and shiny. Metascutellum subrectangular, ventrally concave, strongly coriaceous. Metanotal trough coriaceous, setose, ventral bar of metanotal trough coriaceous, higher than height of metascutellum.

**Figures 6–10.**
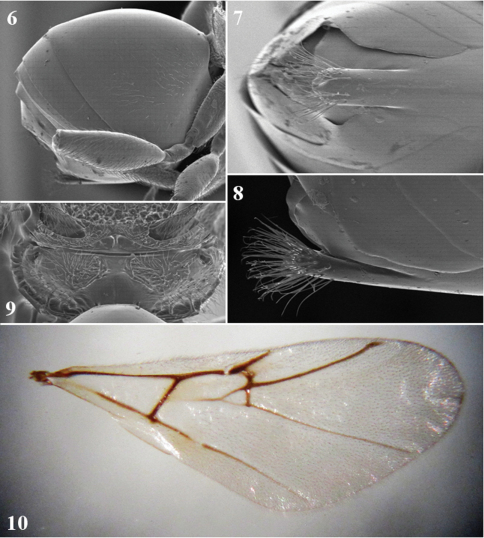
*Coffeikokkos copeyensis*, asexual female **6** metasoma (lateral view) **7** ventral spine of hypopygium (ventral view) **8** ventral spine of hypopygium (lateral view) **9** propodeum **10** forewing.

#### Forewing.

(Fig. 10). 1.35 times longer than body, weakly brown-infuscated, pubescent, with cilia on margins; radial cell open, around 3.5 times as long as broad; veins dark brown; areolet large, triangular, closed.

#### Legs.

 Tarsal claws simple, with broad, rounded basal part (Fig. 4).

#### Metasoma.

(Figs 6–8, 13). As long as head and mesosoma together, longer than high; all metasomal tergites smooth and shiny; 2^nd^ metasomal tergite sparsely setose laterally. Prominent part of ventral spine of hypopygium 2.5 times as long as wide, uniformly broad, with parallel sides, rounded distally, with tuft of long subapical setae, reaching far beyond apex of spine.

**Figures 11–13.**
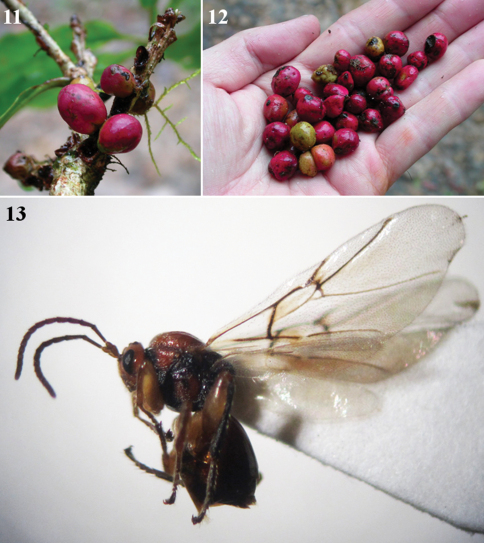
*Coffeikokkos copeyensis*, asexual female **11–12** galls (photos by K. Nishida) **13** habitus, asexual female (lateral view).

#### Gall.

(Figs 11–12). A spherical, slightly ovate stem gall, easily detachable when mature, about 10 mm in diameter; outer surface smooth, shiny, red; with one central larval chamber. Young galls are slightly flattened laterally, yellowish green in colour, and often found in rows along the branch, emerging from an elongated scar in the bark. Mature galls become easily detachable and fall to the leaf litter, where they resemble red coffee fruits before they fall. Adult wasps normally emerge from the galls on the ground.

#### Host plant.

*Quercus bumelioides* Liebm. (Section Quercus of *Quercus*; white oaks), distributed from Mexico to Panama (Govaerts and Frodin 1998).

#### Distribution.

 Currently known only from Costa Rica. Very common species in the Talamanca mountain range of Costa Rica (P. Hanson, personal observation).

#### Biology.

 Only the asexual (parthenogenetic) females are known. Mature galls were collected in September-January, and adults emerged soon after the galls were collected. Further study is necessary to determine the phenology of this species. *Eurytoma* sp., *Sycophila* sp. (both Eurytomidae), and *Torymus* sp. (Torymidae) have been reared from these galls, but no comparisons of parasitization rates have been made with other, sympatric species of Cynipini.

#### Etymology.

 The species is named after the junior synonym of the host plant, *Quercus copeyensis*, on which it induces galls and which name is still in use between the scientists of the given region.

## Discussion

It is essential to make a detailed examination of *Coffeikokkos* diagnostic characters and compare with morphologically similar *Cynips* L. complex-*Atrusca* Kinsey, *Biorhiza* Westwood (particularly with species previously placed in the now synonymized *Sphaeroteras* Ashmead) and *Trigonaspis* Hartig (particularly with species previously placed in the now synonymized *Xanthoteras* Ashmead) –because of one ambiguous character: the state of the tarsal claw. For the newly described genus, we mentioned that the tarsal claws possess a rounded basal lobe. However, this character can be interpreted in two ways: (i) the tarsal claw with a broad rounded basal lobe or (ii) the tarsal claw is simple, with a broad basal part of the claw. It is difficult to define exactly whether a basal lobe is present or the basal part of the claw is just broad (Fig. 4). In all other known Cynipini genera the basal lobe, when present, is acute and a distinct “tooth” is present, while in others the tarsal claw is narrow, without a lobe or (as *Coffeikokkos*) a broadened basal part.

*Coffeikokkos* differs from all known Cynipini genera by antennae that have 14–15 flagellomeres, instead of the usual 11–12. In the diagnosis, we mentioned that *Coffeikokkos* resembles *Cynips* and particularly *Cynips korsakovi*, a species known from Transcaucasus, Azerbaijan (Belizin 1961, Maisuradze 1962) and Iran (G. Melika, personal observation) (see Diagnosis to the genus *Coffeikokkos* above).

All asexual representatives of the entire *Cynips* complex (including synonymised *Antron* Kinsey and *Besbicus* Kinsey; Melika and Abrahamson 2002) have a ligulate, saddle-shaped, 2^nd^ metasomal tergite, the height of the ventral impressed area of the metanotum always shorter that the height of the metascutellum, and the tarsal claws have an acute, distinct basal lobe. There are some species within this complex that resemble *Coffeikokkos*, but in known *Antron* species the mesoscutum is delicately coriaceous or microreticulate, never smooth, glabrous; in many species the prominent part of the ventral spine of the hypopygium is broadened at the apex, and all tergites are laterally densely setose. All *Antron* species are known to induce detachable, usually rounded, leaf galls; galls are never on twigs. Also the *Antron* species known to associate with only white oak species.

In *Atrusca* the antennae have 12 flagellomeres, the mesoscutum is coriaceous or microreticulate, the tarsal claws have an acute basal lobe, the radial cell of the forewing is very short, with Rs strongly curved toward the wing margin and the prominent part of the ventral spine of the hypopygium is very short and broadest at the apex, or long and narrowing toward the apex. The central propodeal area is like that in *Coffeikokkos*, but the lateral propodeal carinae are complete, reaching the nucha, and in the posterior half they are parallel, or only slightly curved outwards; the ventral impressed area is always shorter, sometimes much shorter, than the height of the metascutellum. There are some species of *Atrusca*, e.g., *Atrusca clavuloides* (Kinsey), with a smooth mesoscutum, dull rugose mesoscutellum and the same shape and form of the prominent part of the ventral spine of the hypopygium as in *Coffeikokkos*; however, the antennae have 12 flagellomeres, the mesoscutum is more densely setose, the fore wings have distinct dark spots, and the lateral propodeal carinae are complete, being only slightly curved outwards in the middle, and located much closer to one another. Another species, *Atrusca pulchripennes* (Ashmead), is also similar to *Coffeikokkos* in the polished mesoscutum, the shape of scutellar foveae, and the ventral impressed area of the pronotum is higher than the height of the metascutellum. However, in *Atrusca pulchripenne*, the lower face in anterior view is more transverse, the clypeus is broadly emarginate ventrally (much larger compared to the height of the lower face) and overhangs the mandibles, the radial cell in the forewing is short, with Rs strongly curved backward at the wing margin and the lateral propodeal carinae are complete and much closer to one another in the posterior half; the prominent part of the ventral spine of the hypopygium is needle-like, without a tuft of subapical setae. Moreover, all known species of *Atrusca* (like all asexual forms in the *Cynips* complex) induce galls exclusively on leaves of white oaks.

*Coffeikokkos* also partially resembles another Nearctic genus, the former *Sphaeroteras* (synonymised with *Biorhiza* by Melika and Abrahamson 2002), particularly *Biorhiza rydbergiana* (Cockerell), which has a smooth, sparsely setose mesoscutum along the notauli; it is also similar in the habitus of the mesoscutellum with scutellar foveae, the short, broad, parallel-sided ventral spine of the hypopygium, and the metasoma with sparse setae only on lateral parts of the 2^nd^ metasomal tergite. However, in *Biorhiza rydbergiana* the antennae have 12 flagellomeres; the tarsal claw is simple, without a basal lobe; the ventral impressed area of the metanotum is much shorter than the height of the metascutellum; and the sides of the pronotum, mesopleuron, metascutellum and propodeum have more dense setae. The shape of the central propodeal area somewhat resembles that of *Coffeikokkos* in that the lateral propodeal carinae are parallel and curved outwards in the posterior part of the propodeum; however, they are complete, reaching the nucha, and are slightly less divergent in the posterior half. Galls of *Biorhiza rydbergiana* are induced on leaves.

*Coffeikokkos* also partially resembles yet another Nearctic genus, the former *Xanthoteras* Ashmead (some of the species were synonymised with *Trigonaspis* and others with *Biorhiza* (Melika and Abrahamson 2002)). Some of these species are similar to *Coffeikokkos*, but all have antennae with 12 flagellomeres, tarsal claws with an acute basal lobe, and the malar sulcus is present. In particular, the habitus of *Biorhiza eburnea* (Bassett) and *Biorhiza polita* (Bassett) somewhat resembles that of *Coffeikokkos*: the mesoscutum is polished, with rows of setae along the complete notauli, the mesoscutellum and scutellar foveae are similar in shape and surface sculpture, the shape of the 2^nd^ metasomal tergite, with few lateral setae, is also similar. However, the tarsal claws have an acute basal lobe, the antennae have only 12 flagellomeres, the malar sulcus is present, the lateral propodeal carinae are slightly curved outwards posteriorly, and the ventral impressed area of the metanotum is shorter than the height of the metascutellum, the prominent part of the ventral spine of the hypopygium narrows toward the apex (it is thus more needle-like) and has few subapical setae reaching beyond the apex of the spine, not forming a tuft of setae. Both species of *Biorhiza* induce detachable, spherical leaf galls on white oaks.

There is one distinct feature of *Coffeikokkos*, on the basis of which it is easy to separate this newly described genus from all other known genera: the antennae have 14–15 flagellomeres, while in all other Cynipini only 11–12 are present. Moreover, the very peculiar galls resemble red coffee fruits.

The discovery of the new genus supports the idea about the American radiation centre of Cynipini (Kinsey 1936). Further research and collecting are necessary to decide whether *Coffeikokkos* is an evolutionary novelty distributed only in the Neotropics or the genus representatives are distributed also further northward, into the Nearctic region.

## Supplementary Material

XML Treatment for Coffeikokkos

XML Treatment for Coffeikokkos copeyensis
